# *Curcuma aromatica* Salisb. Protects from Acetaminophen-Induced Hepatotoxicity by Regulating the Sirt1/HO-1 Signaling Pathway

**DOI:** 10.3390/nu15040808

**Published:** 2023-02-04

**Authors:** Hyunseong Kim, Jinyoung Hong, Junseon Lee, Wanjin Jeon, Changhwan Yeo, Yoonjae Lee, Seungho Baek, Inhyuk Ha

**Affiliations:** 1 Jaseng Spine and Joint Research Institute, Jaseng Medical Foundation, Seoul 06110, Republic of Korea; 2 College of Korean Medicine, Dongguk University, 32 Dongguk-ro, Ilsandong-gu, Goyang-si 10326, Republic of Korea

**Keywords:** *Curcuma aromatica* Salisb., acetaminophen, paracetamol, hepatotoxicity, Sirt1, HO-1

## Abstract

Acetaminophen (APAP) overdose-induced hepatotoxicity reduces the activity of sirtuin-1 (Sirt1) along with heme oxygenase 1 (HO-1) and promotes inflammatory responses and oxidative stress. Although the extract of *Curcuma aromatica* Salisb. (CAS) possesses hepatoprotective properties, scientific evidence on whether CAS prevents hepatotoxicity and the underlying molecular mechanisms are lacking. Here, we hypothesized that CAS ameliorates hepatotoxicity by inhibiting inflammation and oxidative stress via Sirt1/HO-1 signaling. CAS pretreatment at doses of 200 and 400 μg/mL significantly increased cell viability in APAP-treated primary hepatocytes. The expression of inducible nitric oxide synthase (iNOS) substantially increased after APAP treatment; however, this expression significantly decreased in cells pretreated with 100, 200, and 400 µg/mL CAS. CAS increased Sirt1 and HO-1 levels in APAP-treated hepatocytes in a dose-dependent manner. When CAS was orally administered to mice at doses of 20 or 100 mg/kg for 7 days, the APAP-induced increase in serum aspartate aminotransferase and alanine aminotransferase levels was inhibited. Moreover, CAS decreased IL-6, TNF-α, and IL-1β, increased IL-10, suppressed ROS generation, increased glutathione levels, inhibited iNOS and cyclooxygenase-2, and enhanced Sirt1 and HO-1 in the mouse model of APAP-induced hepatotoxicity. These findings suggest that CAS could be used as a natural hepatoprotective drug to treat APAP-induced injury.

## 1. Introduction

Acetaminophen (APAP) is a widely used antipyretic/analgesic agent. Overdosage causes hepatotoxicity, which leads to acute liver failure [1,2]. It has been reported that most cases of acute hepatotoxicity occurring in the United States are due to APAP, which is a major cause of severe liver damage requiring liver transplantation [3,4]. APAP toxicity is primarily caused by hydrogen peroxide and superoxide produced during metabolic processes [5]. Peroxides, which are generated under oxidative stress, are representative products of reactive oxygen species that promote tissue damage and inflammatory responses in the liver [6,7]. Thus, reducing oxidative stress is a fundamental therapeutic option for the treatment of APAP-induced hepatotoxicity. Many natural health products effectively protect against a variety of oxidative stress-related diseases such as cardiovascular disease, cancer, and liver disease [8,9].

*Curcuma aromatica* Salisb. (CAS) is distributed throughout India, China, and Japan and is a perennial plant of the Zingiberaceae that grows wild in hot and humid southern Asia, Africa, and Central and South America [10]. Phytochemical constituents of CAS such as tumerone, curlone, curcuphenol, curcumin, demethoxycurcumin, bisdemethoxycurcumin, and cyclocurcumin possess wound healing, antioxidative, antimicrobial, anti-inflammatory, and antitumor properties [11,12]. In particular, curcumin, the major component of CAS, is contained at 2–5% of dry mass [13] and has strong antibacterial, antioxidant, anti-inflammatory, and anticancer properties [14]. Therefore, CAS is used as a health food in herbal compositions for the prevention and treatment of liver diseases [15,16]. Saengkan-hwan, in which CAS is the main ingredient, is used in Korean hospitals to treat liver damage. However, scientific evidence of the protective effects of CAS in hepatocytes is still insufficient. Furthermore, the detailed underlying molecular mechanisms whereby CAS prevents hepatotoxicity remain unclear.

Recent studies have reported that the health benefits of sirtuin 1 (Sirt1) include hepatoprotective effects [17,18]. Sirt1 is an NAD^+^-dependent histone deacetylase with functions related to DNA repair, tissue regeneration, cell survival, inflammation, neuronal signaling, and circadian rhythms [19,20]. APAP reduces Sirt1 activity, which activates IL-1β/NF-κB signaling and leads to enhanced inflammation and oxidative damage, causing significant liver and hepatocyte damage both in vivo and in vitro [21,22]. The promotion of Sirt1 activity ameliorates oxidative stress and inflammation responses, potentially alleviating liver-mediated APAP hepatotoxicity [23]. Furthermore, Sirt1 is a redox-sensitive protein deacetylase that plays an important role in intracellular redox homeostasis, along with well-known antioxidants such as heme oxygenase 1 (HO-1) [24].

Here, we hypothesized that CAS ameliorates APAP-induced hepatotoxicity by inhibiting inflammation and oxidative stress via the activation of Sirt1/HO-1 signaling in mice and sought to test this hypothesis. To the best of our knowledge, this is the first study to demonstrate the in vitro and in vivo hepatoprotective effects of CAS against APAP hepatotoxicity.

## 2. Materials and Methods

### 2.1. Primary Mouse Hepatocytes

Primary hepatocytes were obtained with the standard two-step collagenase perfusion procedure via portal vein perfusion as previously described [25]. Briefly, C57BL/6 mice (7–8 weeks old, Daehan Bio Link, Eumseong, Chungbuk, Korea) were anesthetized by gas inhalation of 2–3% isoflurane gas (Forane; BK Pham, Goyangsi, Korea) and underwent laparotomy. The liver was perfused from the hepatic portal vein and vena cava with 50 mL of the Liver Perfusion Medium (Gibco, Grand Island, NY, USA) and cannulated using 50 mL of the Liver Digest Medium (Gibco) pre-heated to 37 °C. The liver tissue was extracted and ruptured with fine-tip forceps, and then released using a cell lifter (SPL Lifesciences, Pocheon, Korea). The collected tissue lysates were filtered by a 70 μm cell strainer (miltenyl, Teterow, Germany) and purified using 90% (*v*/*v*) percoll (Sigma, St Louis, MO, USA) and density gradient centrifugation. The cells were seeded into each well in collagen type I-coated 24-well plates (Gibco) at a density of 1 × 10^5^ cells/well (6-well plate; 5  ×  10^5^ cells/well). All animal experiments were approved by the Jaseng Animal Care and Use Committee (approval number: JSR-2021-10-007-A).

### 2.2. Preparation of CAS Extract

CAS extract was prepared as described previously [26]. The dried root of 30 g CAS was boiled at 105 °C with 300 mL distilled water for 3 h, cooled on ice, and then stored in a deep freezer. Next, a lyophilized extract was obtained in a freeze dryer (Ilshin BioBase, Yangju, Korea). The CAS powder was dissolved in phosphate-buffered saline (PBS) to prepare a 10 mg/mL density.

### 2.3. APAP-Induced Liver Injury and CAS Treatment

Hepatocytes were cultured in 96-well plates in William’s E medium (Gibco) with the addition of L-glutamine (2 mmol/L) and 1% P/S for 24 h. Then, the hepatocyte culture medium was replaced with a medium containing different concentrations of CAS (1, 10, 25, 50, 100, 200, and 400 μg/mL) for the Cell Counting Kit-8 (CCK-8) assay. After a 30 min treatment, APAP was added at 2 mM to the culture medium containing the CAS and then incubated in 5% CO_2_ at 37 °C for 24 h. To perform the CCK assay, CCK was applied at a final concentration in a culture medium of 10% at 37 °C for 4 h. Then, optical density was measured by a microplate reader (Epoch, BioTek, Winooski, VT, USA) at 450 nm. The cell viability was expressed as the mean percentage values of viable hepatocytes relative to the blank group.

### 2.4. Flow Cytometry

ROS levels were assessed using 2,7-dichlorofluorescin diacetate (DCFDA; Sigma Aldrich). Briefly, we treated cell pellets with a 10 µM DCFDA solution prepared from a 5 mM stock solution. Relative differences between groups were expressed as the percentage of the values relative to the mean of the APAP group.

### 2.5. Immunocytochemistry

Cells were cultured and divided into five groups as follows; (1) Blank (no treatment), (2) APAP only, (3) 100 µg/mL of CAS pretreated + APAP, (4) 200 µg/mL of CAS pretreated + APAP, and (5) 400 µg/mL of CAS pretreated + APAP. After culturing under group conditions, washed cells were incubated with 4% paraformaldehyde for at least 30 min and treated with 0.2% Triton X-100 for 10 min. Then, cells were stained with the following diluted antibodies in 2% normal goat serum (NGS) for 1 h: iNOS (1:100; Cell signaling, Beverly, MA, USA), DAPI (1:1000; Tokyo Chemical Industry Co, Japan), Sirt1 (1:100; Abcam, Cambridge, UK), and HO-1 (1:500; Abcam). FITC, Rhodamine Red-X, Alexa Fluor^®^ 594, 647 recognizing rabbit, mouse, and guinea pig IgG were added to the 2% NGS at 1:300 for 2 h at room temperature and washed three times with PBS. DAKO Fluorescent mounting media was treated on cells to preserve fluorescence, and 10 areas were captured at 100× or 400× magnification on a confocal microscope (Eclipse C2 Plus, Nikon, Tokyo, Japan). Measurement of mean fluorescence intensity was performed with ImageJ software and compared to the APAP group.

### 2.6. In Vivo Model of APAP-Induced Liver Injury

Male C57BL/6 mice (7 weeks old, 20 ± 2 g) were used for evaluating CAS protective efficacy and safety against APAP-induced liver injury. Animal experiments were conducted in accordance with the animal ethics guidance approved by the Jaseng Animal Care and Use Committee (JSR-2021-10-007-A). Mice were housed at a constant temperature (25 ± 1 °C) and humidity (65 ± 5%) on a 12 h light/dark cycle. Forty-eight mice were randomly divided into six groups of eight mice each as follows: (1) Normal group (Normal), (2) 20 mg/kg CAS (CAS20), (3) 100 mg/kg CAS (CAS100), (4) 300 mg/kg acetaminophen (APAP), (5) 20 mg/kg CAS + APAP (CAS20 + APAP), and (6) 100 mg/kg CAS + APAP (CAS100 + APAP). APAP was intraperitoneally injected at a dose of 300 mg/kg on the day after the last CAS injection. Meanwhile, CAS was administered orally at a dose of 20 or 100 mg/kg once daily for 1 week. Body weight was measured on days 1, 3, 5, 7, and 9. The rate of weight gain = last-day weight (g)/first-day weight (g) × 100% − 100. The blood and liver were separated and stocked at 24 h after the APAP injection.

### 2.7. Serum Analyses

Liver function was confirmed by analyzing the alanine aminotransferase (ALT) and aspartate transaminase (AST) levels. The blood was drawn using an intracardiac method and serum was separated by centrifugation at 3,000 rpm for 10 min at 4 °C. Serum ALT and AST levels were calculated by an automated dry chemistry analyzer (FUJI DRI-CHEM 7000i, Tokyo, Japan).

### 2.8. Histology

To confirm the liver injury, liver tissues were dehydrated, cleared, and embedded in paraffin. Paraffin blocks were sectioned in the coronal plane at 16 µm to get 10 µm sliced samples. In order to confirm the degree of liver damage, the central portion of the sectioned slides was stained with a hematoxylin and eosin solution and captured with an inverted microscope (Nikon, Tokyo, Japan).

### 2.9. ELISA

The inflammation and oxidative stress-involved factors (IL-6, IL-1β, TNF-α, and IL-10, ROS, and GSH) were measured by ELISA kits (BD Biosciences, Franklin Lakes, NJ, USA and Abcam, Cambridge, UK) according to the manual of the manufacturer. Briefly, the radioimmunoprecipitation assay buffer (GenDEPOT, Barker, TX, USA) containing a proteinase inhibitor (Millipore, Billerica, MA, USA) was treated for extracting liver tissues using a tacoTM Prep Bead Beater (GeneReach, Taichung, Taiwan) and collected by centrifugation at 13,000 rpm at 4 °C for 10 min. Measurement of the protein concentration was accomplished using a bicinchoninic acid protein assay kit (Thermo Fisher Scientific, Waltham, MA, USA).

### 2.10. Reverse Transcription Quantitative PCR (RT-qPCR)

Changes in liver mRNA expression of IL-6, TNF-α, IL-1β, IL-10, iNOS, COX2, Sirt1, and HO-1 genes were measured using RT-qPCR. Total RNA was isolated by treating the liver with the TRIzol reagent (Ambion, Austin, TX, USA), and the RNA template was converted to cDNA using the oligo(dT)20 primer and Accupower RT PreMix (Bioneer, Daejeon, Korea). The RT-qPCR was performed using iQ SYBR Green Supermix (Bio-Rad) on a CFX Connect Real-Time PCR Detection System (Bio-Rad) under cycling conditions as follows: 40 cycles at 95 °C for 3 min, 95 °C for 15 s, and 60 °C for 30 s. Six different samples were analyzed in triplicate and expressed as the fold-change relative to the APAP group by normalization to GAPDH. The primers used for RT-qPCR are listed in Table 1.

### 2.11. Statistics

The results are expressed as the mean ± standard error of the mean (SEM). Statistical comparisons were performed using one-way or two-way ANOVA with Tukey’s post- hoc analysis (Graph-Pad Prism 8.0, Inc., La Jolla, CA, USA). Statistical significance was set at # *p* < 0.05, ## *p* < 0.01, ### *p* < 0.001, and #### *p* < 0.0001 vs. the blank group and * *p* < 0.05, ** *p* < 0.01, *** *p* < 0.001, and **** *p* < 0.0001 vs. the APAP group.

## 3. Results

### 3.1. CAS Suppresses APAP-Induced Cell Viability Reduction and ROS Generation in Primary Mouse Hepatocytes

**Figure 1:**
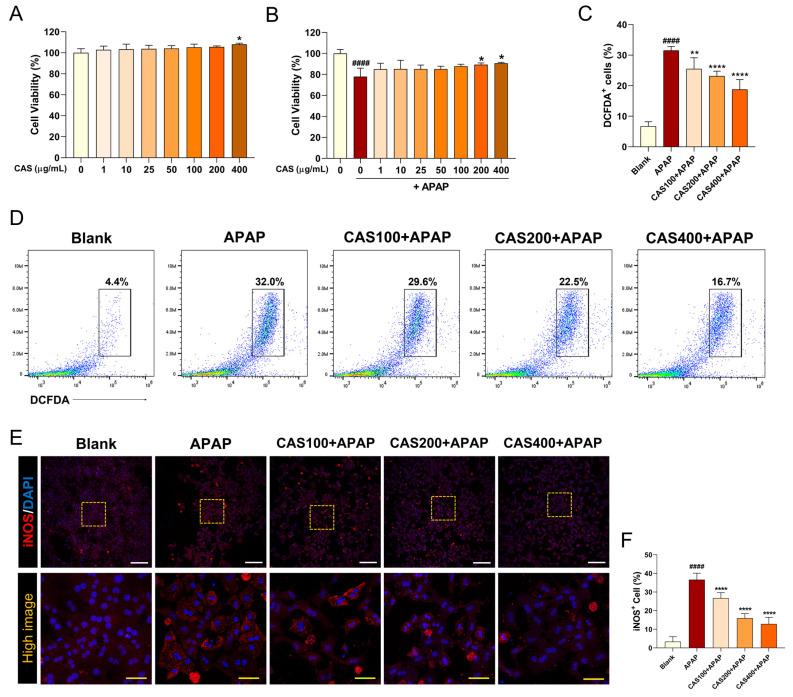
CAS protects primary mouse hepatocytes from APAP-induced hepatotoxicity and oxidative damage by modulating ROS production and iNOS expression. Determination of cell viability in primary hepatocytes pretreated with CAS for 24 h (**A**) without APAP treatment and (**B**) with APAP treatment using a cell counting kit assay (*n* = 4). (**C**) Flow cytometric quantification of the percentage of DCFDA^+^ cells upon challenge with APAP (*n* = 6). (**D**) Representative flow cytometric dot plot images showing DCFDA-FITC analysis for intracellular ROS levels. (**E**) Representative confocal images of immunocytochemistry for iNOS (red) in the primary hepatocytes of each group. White scale bar = 200 µm, yellow scale bar = 50 µm. (**F**) Quantitative analysis of the percentage of iNOS-positivity in DAPI-positive hepatocytes after pretreatment with CAS at 100, 200, and 400 µg/mL and treatment with 2 μM APAP (*n* = 6). The data are expressed as the mean ± SEM and were analyzed via one-way analysis of variance with Tukey’s post-hoc test. Significant differences are indicated as follows: #### *p* < 0.0001 vs. blank group; * *p* < 0.05, ** *p* < 0.01, and **** *p* < 0.0001 vs. APAP group.

To investigate whether CAS treatment can ameliorate the APAP-induced reduction in cell viability, we performed a CCK-8 assay in primary mouse hepatocytes after 24 h of treatment with CAS and APAP. CAS was not toxic to cultured hepatocytes at concentrations of up to 400 µg/mL and significantly increased cell viability at 400 µg/mL (Figure 1A). In addition, the effective dose of CAS for hepatoprotection was determined in hepatocytes exposed to APAP. Cell viability after APAP exposure was decreased significantly compared with that in the blank group. In contrast, CAS significantly increased cell viability at 200 and 400 µg/mL (Figure 1B). We examined whether CAS had an antioxidant effect through the inhibition of APAP-induced ROS generation using FACS analysis. Exposure to APAP significantly increased ROS generation, whereas pretreatment with CAS significantly reduced ROS generation in a dose-dependent manner (Figure 1C,D). After confirming that CAS pretreatment induced a decrease in ROS production in APAP-treated hepatocytes, we also examined inducible nitric oxide synthase (iNOS) expression using immunocytochemistry. When APAP was used, the percentage of iNOS-positive cells increased compared with that in the blank group, in which the percentage was <10. CAS pretreatment resulted in a dose-dependent decrease in the percentage of iNOS-positive cells in APAP-treated hepatocytes (Figure 1E,F).

### 3.2. CAS Protects Cultured Hepatocytes from APAP Hepatotoxicity by Activating the Sirt1/HO-1 Signaling Pathway

**Figure 2:**
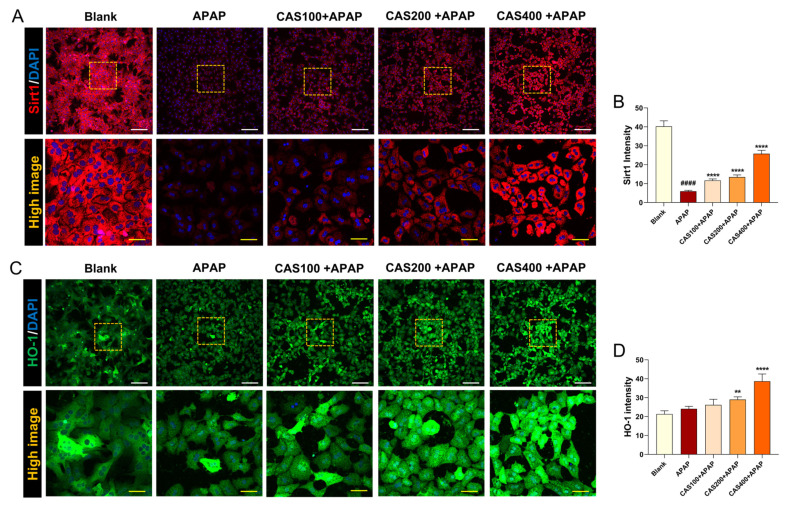
The hepatoprotective effects of CAS on APAP-induced hepatotoxicity involve the upregulation of Sirt1/HO-1 expression in primary mouse hepatocytes. (**A**) Representative confocal images of immunocytochemistry for Sirt1 (red) in the blank, APAP, and CAS groups. White scale bar = 200 µm, yellow scale bar = 50 µm. (**B**) Quantitative analysis of the relative fluorescence intensity of Sirt1 in each group (*n* = 6). (**C**) Representative confocal images of immunocytochemistry for HO-1 (green) in the blank, control, and CAS groups. White scale bar = 200 µm, yellow scale bar = 50 µm. (**D**) Quantitative analysis of the relative fluorescence intensity of HO-1 in each group (*n* = 6). The data are expressed as the mean ± SEM and were analyzed via ordinary ANOVA with Tukey’s post-hoc analysis. Significant differences are indicated as follows: #### *p* < 0.0001 vs. blank group; ** *p* < 0.01 and **** *p* < 0.0001 vs. APAP group.

We further evaluated changes in the expression levels of Sirt1 and HO-1, which are involved in hepatoprotection, through an immunochemistry assay. Sirt1 was strongly expressed in untreated hepatocytes, but its expression was significantly suppressed after APAP treatment. In contrast, Sirt1 immunoreactivity significantly increased in a dose-dependent manner when the cells were pretreated with CAS (Figure 2A). Quantitatively, CAS pretreatment increased Sirt1 intensity in a dose-dependent manner (Figure 2B). In addition, the Sirt1 inhibitor EX-527 was added to block Sirt1 signaling, and cell viability was assayed using the CCK-8 assay. EX-527 treatment abrogated the protective effect of CAS after APAP treatment (Supplementary Figure S2). Thus, we demonstrated that the hepatoprotective effect of CAS is closely related to the Sirt1 signaling pathway. We also analyzed changes in HO-1 expression to investigate whether the antioxidant effects of CAS on APAP-induced oxidative stress involve HO-1. In confocal microscopy images, APAP treatment did not affect the expression levels of HO-1, whereas CAS pretreatment increased HO-1 levels (Figure 2C). Quantitative analysis of the relative fluorescence intensity showed that CAS increased HO-1 expression in a dose-dependent manner with the expression peaking at 400 µg/mL CAS (Figure 2D).

### 3.3. CAS Protects the Liver from APAP-Induced liver Injury in Mice

**Figure 3:**
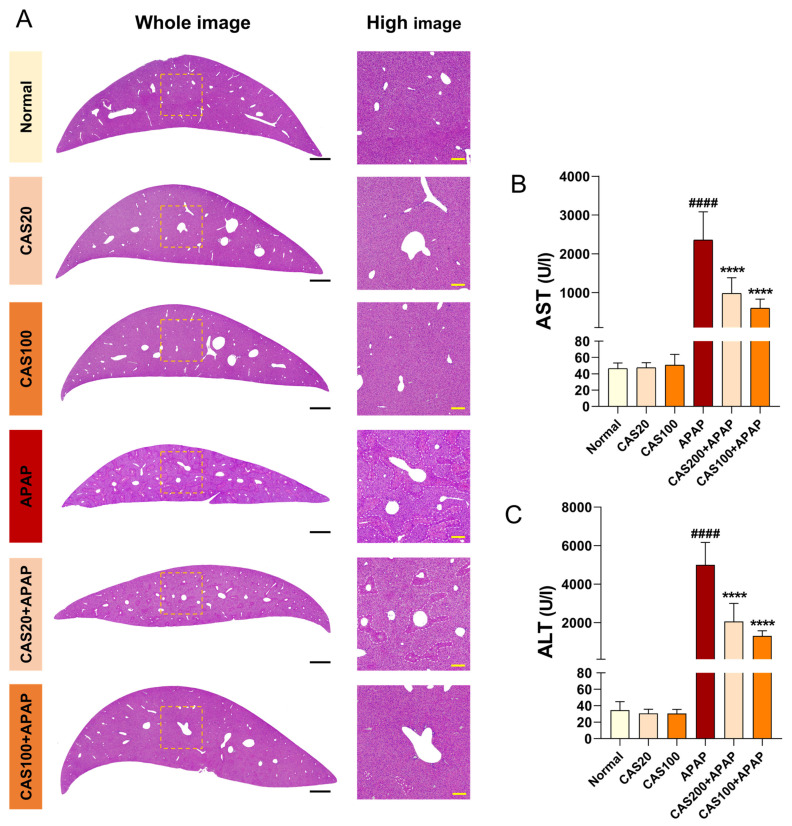
Prevention of histopathological liver damage by CAS administration in a mouse model of APAP-induced hepatotoxicity. (**A**) Representative H&E images of liver structural damage in the blank, control, and CAS groups. White scale bar = 1000 µm, yellow scale bar = 100 µm. (**B**,**C**) Quantitative analysis of the serum AST and ALT levels in blood from each group of mice (*n* = 8). The data are expressed as the mean ± SEM and were analyzed via ordinary ANOVA with Tukey’s post-hoc analysis. Statistical differences are indicated as follows: #### *p* < 0.0001 vs. blank group; **** *p* < 0.0001 vs. APAP group.

To evaluate the extent of hepatoprotection exerted on CAS administration in a mouse model of APAP-induced hepatotoxicity, we performed hematoxylin and eosin (H&E) staining of liver tissue after CAS and APAP administration. H&E-stained sections showed necrotic patterns after APAP application. However, minimal morphological changes, characterized by hepatocyte necrosis, were observed in the CAS-treated group (Figure 3A). In addition, the serum aspartate aminotransferase (AST) and alanine aminotransferase (ALT) levels were abnormally elevated after APAP administration. However, CAS treatment significantly reduced the levels of these enzymes in the mouse model of APAP toxicity (Figure 3B,C). These findings demonstrated that CAS ameliorates liver damage and elevated plasma ALT/AST enzyme levels caused by APAP overdose.

### 3.4. CAS Ameliorates APAP-Induced Liver Inflammation in Mice

**Figure 4:**
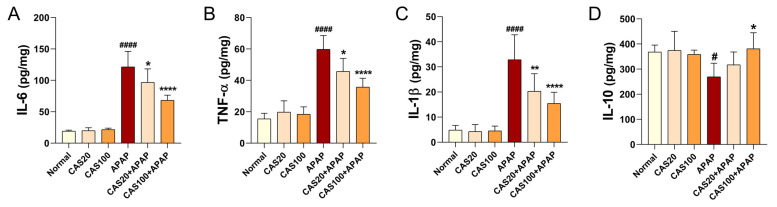
Anti-inflammatory activity of CAS in APAP-induced hepatic inflammation in mice. (**A**–**C**) ELISA results showing the expression of the inflammation-related cytokines, (A) IL-6, (B) TNFα, and (**C**) IL-1β in the liver tissues of each group (*n* = 6). (**D**) ELISA results of IL-10 expression in the liver tissues of each group (*n* = 6). The data are expressed as the mean ± SEM and were analyzed via one-way ANOVA with Tukey’s post-hoc test. Statistical differences are indicated as follows: # *p* < 0.05 and #### *p* < 0.0001 vs. blank group; * *p* < 0.05, ** *p* < 0.01, and **** *p* < 0.0001 vs. APAP group.

ELISA was performed on liver tissues to detect changes in protein expression associated with the inflammatory response after CAS administration in the mouse model of APAP-induced hepatotoxicity. The expression levels of inflammation-related cytokines, including IL-6, TNF-α, and IL-1β, were significantly higher in the APAP group than in the normal group. CAS administration significantly decreased the levels of these cytokines in a dose-dependent manner (Figure 4A–C). In contrast, the expression level of IL-10, a potent anti-inflammatory cytokine, significantly decreased in the APAP group compared with that in the normal group, and IL-10 levels dose-dependently increased on CAS administration. However, the increase was only statistically significant in the 100 mg/kg CAS group (Figure 4D). The CAS extract suppressed IL-6, TNF-α, and IL-1β expression while promoting IL-10 expression in the mouse model of APAP-induced hepatotoxicity.

### 3.5. CAS Alleviates APAP-Induced Oxidative Damage and Glutathione Depletion by Activating Sirt1/HO-1 mRNA Expression in Mice

**Figure 5:**
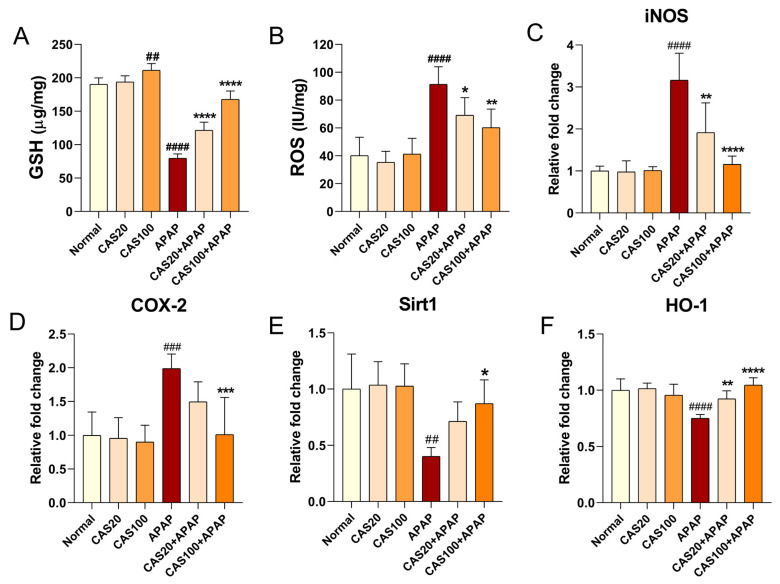
Hepatic antioxidant effect of CAS against APAP-induced oxidative stress in mice via modulating Sirt1/HO-1 mRNA expression. (**A**) The hepatic GSH content in the liver tissues of each group (*n* = 6). (**B**) The ROS level in the liver tissues of each group (*n* = 6). (**C**–**F**) RT-qPCR results of antioxidant-related mRNA expression (*iNOS, COX-2, Sirt1*, and *HO-1*) in the liver tissues of mice after CAS and APAP administration (*n* = 6). The data are expressed as the mean ± SEM and were analyzed via one-way ANOVA with Tukey’s post-hoc test. Significant differences are indicated as follows: ## *p* < 0.01, ### *p* < 0.001, and #### *p* < 0.0001 vs. blank group; * *p* < 0.05, ** *p* < 0.01, *** *p* < 0.001, and **** *p* < 0.0001 vs. APAP group.

To investigate the antioxidant effect of CAS on APAP-induced oxidative damage, we examined total glutathione (GSH) and ROS levels in liver tissues. Total GSH levels in the liver tissues of mice treated with APAP significantly decreased; CAS administration increased GSH levels in the liver in a dose-dependent manner (Figure 5A). Moreover, CAS significantly inhibited APAP-induced ROS accumulation in the liver (Figure 5B). We performed RT-qPCR analysis of the liver tissue to quantify changes in antioxidant gene expression, including *iNOS*, *COX2*, *Sirt1*, and *HO-1*, after CAS administration in the APAP-induced hepatotoxicity model. The expression of *iNOS* and cyclooxygenase-2 (*COX-2*) was significantly upregulated in the APAP group compared with that in the normal group, whereas *iNOS* expression was significantly downregulated in the CAS group compared with that in the APAP group (Figure 5C). *COX-2* expression was also dose-dependently downregulated in the groups treated with CAS extracts, but the difference between the CAS and APAP groups was only statistically significant when CAS was used at 100 mg/kg CAS (Figure 5D). Moreover, the gene expression of *Sirt1* and *HO-1,* which are involved in antioxidant activity, was significantly downregulated in the APAP group compared with that in the normal group. In contrast, CAS dose-dependently increased *Sirt1* expression, but the increase was statistically significant only in the 100 mg/kg CAS group (Figure 5E). CAS administration dose-dependently increased *HO-1* expression compared with the control (Figure 5F).

## 4. Discussion

Excessive long-term drug intake can cause tissue toxicity and damage, which frequently occurs in the liver because it breaks down drugs. The most representative drug causing liver toxicity is APAP [25]. The liver is one of the most resilient tissues and is capable of self-regenerating; however, drug misuse and abuse can cause severe toxicity and lead to liver damage [26]. Many traditional herbal medicines have been used to treat human diseases since ancient times, but their potential risk for hepatotoxicity has been considered a serious medical issue [27]. However, CAS has excellent hepatoprotection effects, effectively ameliorating hepatotoxicity by promoting bile secretion [28]. Furthermore, curcumin, a yellow polyphenolic pigment that is the main constituent of CAS, has therapeutic and protective effects on oxidation-related liver diseases [14,29]. The phenol structure in curcumin is known to scavenge oxygen-derived free radicals such as the hydroxyl radical, singlet oxygen, superoxide radical, nitrogen dioxide, and NO [30]. In particular, previous relevant studies on the protective effects of APAP-induced liver injury showed that curcumin inhibits APAP-induced hepatotoxicity together with N-acetyl cysteine (NAC) and suppresses ROS by inducing GSH, similar to the effects of CAS in our results [31,32]. When primary hepatocytes were treated with NAC and then subjected to APAP damage, NAC treatment increased cell viability more effectively than CAS in a concentration-dependent manner (Supplementary Figure S3). The mechanisms of action include the inhibition of oxidative stress-related inflammation via NF-κB signaling [33] and the amelioration of liver oxidative stress through the induction of antioxidant signaling pathways such as Nrf2 upregulation [34] and upregulation of antioxidant gene expression (e.g., NQO1, HO-1) through ERK/p38-MAPK pathways [35,36]. The activation of HO-1 may play an important role in the free radical scavenging activity of curcumin [37,38]. Therefore, CAS has been utilized to develop liver health/functional food products. However, the appropriate hepatoprotective dose of CAS at which hepatotoxicity is not induced has not been investigated. In addition, the molecular mechanisms whereby CAS may prevent APAP-induced hepatotoxicity remain unknown.

Here, we hypothesized that CAS ameliorates hepatotoxicity by inhibiting inflammation and oxidative stress via Sirt1/HO-1 signaling and sought to test this hypothesis. We demonstrate that up to 400 µg/mL CAS does not cause toxicity to primary hepatocytes, and that CAS effectively promotes hepatocyte survival by inhibiting APAP-induced hepatic ROS formation and iNOS expression. Interestingly, the protective effect of CAS against APAP toxicity in hepatocytes was achieved via the activation of the HO-1/Sirt1 signaling pathway. Previous studies demonstrated that HO-1-mediated increase in Sirt1 expression plays a crucial role in hepatoprotection against APAP-induced hepatotoxicity by modulating inflammation and oxidative stress [39,40]. We further confirmed the hepatoprotective effect of CAS through the HO-1-mediated increase in Sirt1 expression in a mouse model of APAP-induced hepatotoxicity. First, the degree of liver damage caused by APAP and CAS administration was confirmed by the histological staining of liver tissue. The APAP challenge led to hepatic necrosis around blood vessels, but CAS administration reduced the necrosis of hepatocytes in liver tissue. Liver function tests usually measure the levels of AST and ALT in serum [41]. APAP administration resulted in elevated AST and ALT levels, and these were reduced by CAS treatment. In addition, toxicologically significant weight loss was observed upon APAP administration, whereas administration of CAS alone to normal rats for one week resulted in a steady body weight increase. Therefore, CAS did not cause any hepatotoxicity.

APAP also caused a dramatic increase in hepatic ROS action and a decrease in GSH content. Normally, APAP is metabolized in the liver and converted into a toxic intermediate called N-acetyl-p-benzoquinone imine (NAPQI), which is then quickly conjugated with GSH and released into the blood and out of the body [42]. However, excessive NAPQI produced by an APAP overdose depletes GSH and promotes ROS production, resulting in hepatocellular damage and necrosis [5]. It has also been reported that GSH depletion causes hepatotoxicity by increasing TNF-α gene expression [43]. In this study, toxic doses of APAP decreased GSH activity, whereas CAS treatment increased GSH activity. It should be noted that GSH content was significantly increased even when CAS alone was administered. This means that CAS may offer liver protection even in the absence of hepatotoxicity.

Our findings present the first evidence of the involvement of Sirt1 and HO-1 in the protective and antioxidant effects of CAS against APAP-induced liver toxicity in mice. Oxidative stress and its inhibition are important processes in the development and recovery from hepatotoxicity, respectively. In APAP-induced oxidative stress, excessive NAPQI induces mitochondrial dysfunction, a major cause of ROS production, resulting in nuclear DNA fragmentation, hepatocyte death, and inflammatory responses, including pro-inflammatory cytokine and immune cell activation. NO produced by iNOS promotes liver damage by interfering with mitochondrial respiration [44]. Our results showed that iNOS expression increases after APAP administration in primary hepatocytes and decreases when the cells are pretreated with CAS. Furthermore, the administration of APAP to mice increased ROS levels, the mRNA expression of *iNOS* and *COX2*, and the protein expression of the pro-inflammatory cytokines TNF-α, IL-6, and IL-1b. CAS treatment reversed the deteriorating condition in APAP-exposed mice. Specifically, CAS increased the protein expression of Sirt1 and HO-1 in a dose-dependent manner in primary hepatocytes and increased the mRNA expression of these genes in liver tissue.

Our study has limitations because histological evaluation is insufficient. Therefore, further studies are required to confirm that the hepatoprotective effects of CAS are mediated via Sirt1 and HO-1 signaling. Mechanistic studies using hepatocyte-specific Sirt1 and HO-1 knockout mice can be considered.

## 5. Conclusions

In conclusion, we found that CAS ameliorates APAP-induced liver injury, inflammation, and oxidative damage in mice. In addition, we demonstrated that the hepatoprotective activity of CAS is associated with Sirt1/HO-1 expression. To the best of our knowledge, this is the first study to demonstrate the in vitro and in vivo hepatoprotective effects of CAS against APAP hepatotoxicity and to elucidate the mechanisms whereby CAS exerts these effects. Our findings suggest that CAS may potentially be used to treat APAP-induced liver damage.

## Figures and Tables

**Table 1:** Primer sequences used for RT-qPCR analysis.

Gene	5′-3′	Primer Sequence
*IL-6*	Forward	CCACCCACAACAGACCAGTA
	Reverse	GGAACTCCAGAAGACCAGAGC
*TNF-α*	Forward	CCGACTACGTGCTCCTCACC
	Reverse	CTCCAAAGTAGACCTGCCCG
*IL-1β*	Forward	TTGCTTCCAAGCCCTTGACT
	Reverse	GGTCGTCATCATCCCACGAG
*IL-10*	Forward	TAACTGCACCCACTTCCCAG
	Reverse	AGGCTTGGCAACCCAAGTAA
*iNOS*	Forward	ATGGCTTGCCCCTGGAAGTT
	Reverse	TGTTGGGCTGGGAATAGCAC
*COX2*	Forward	CTCAGCCATGCAGCAAATCC
	Reverse	GGGTGGGCTTCAGCAGTAAT
*Sirt1*	Forward	AGGGAACCTCTGCCTCATCT
	Reverse	TGGCATACTCGCCACCTAAC
*HO-1*	Forward	CCCACCAAGTTCAAACAGCTC
	Reverse	AGGAAGGCGGTCTTAGCCTC
*GAPDH*	Forward	CCCCCAATGTATCCGTTGTG
	Reverse	TAGCCCAGGATGCCCTTTAGT

## Data Availability

Data are contained within the article and Supplementary Material.

## Supplementary Materials

The following supporting information can be downloaded at: https://www.mdpi.com/article/10.3390/nu15040808/s1, Figure S1: Rate of weight gain (%) in mice orally injected with acetaminophen (APAP group), or CAS at two concentrations (20, or 100 μg/kg) in the presence of APAP (*n* = 6). Figure S2: Cell viability of primary hepatocytes pretreated with CAS (100, 200, and 400 μg/mL) followed by subsequent exposure to Sirt1 inhibitor EX-527 with APAP for 24 h and determined by CCK-8 (*n* = 4). Figure S3: Cell viability of primary hepatocytes pretreated with CAS (100, 200, and 400 μg/mL) followed by subsequent exposure to ROS inhibitor NAC (100, 200, and 400 μM) with APAP for 24 h and determined by CCK-8 (*n* = 4).
