# Supplementary material for: Curcuma aromatica Salisb. Protects from Acetaminophen-Induced Hepatotoxicity by Regulating the Sirt1/HO-1 Signaling Pathway
Source: Nutrients. 2023 Feb 4;15(4):808. doi: 10.3390/nu15040808 (PMC9964786; doi:10.3390/nu15040808)
Supplement: Supplementary file 1 [file nutrients-15-00808-s001.zip › nutrients-2145707-supplementary.pdf]

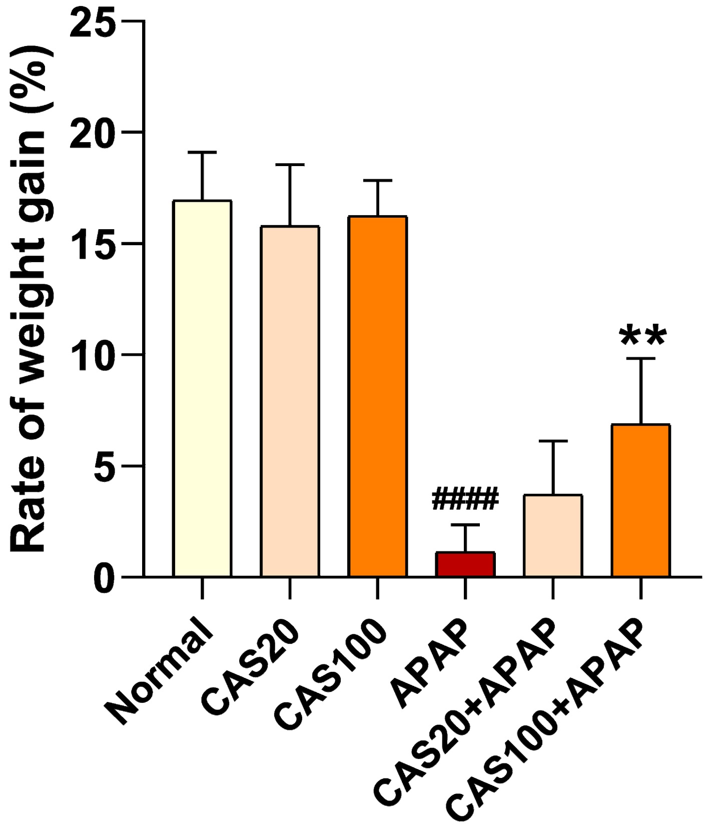

Figure S1. Rate of weight gain (%) in mice orally injected with acetaminophen (APAP group), or CAS at two concentrations (20, or 100 μg/kg) in the presence of APAP ($n = 6$). The data are expressed as the mean ± SEM and were analyzed via one-way analysis of variance with Tukey's post-hoc test. Significant differences are indicated as follows: #### $p < 0.0001$ vs. normal group; ** $p < 0.01$ vs. APAP group.

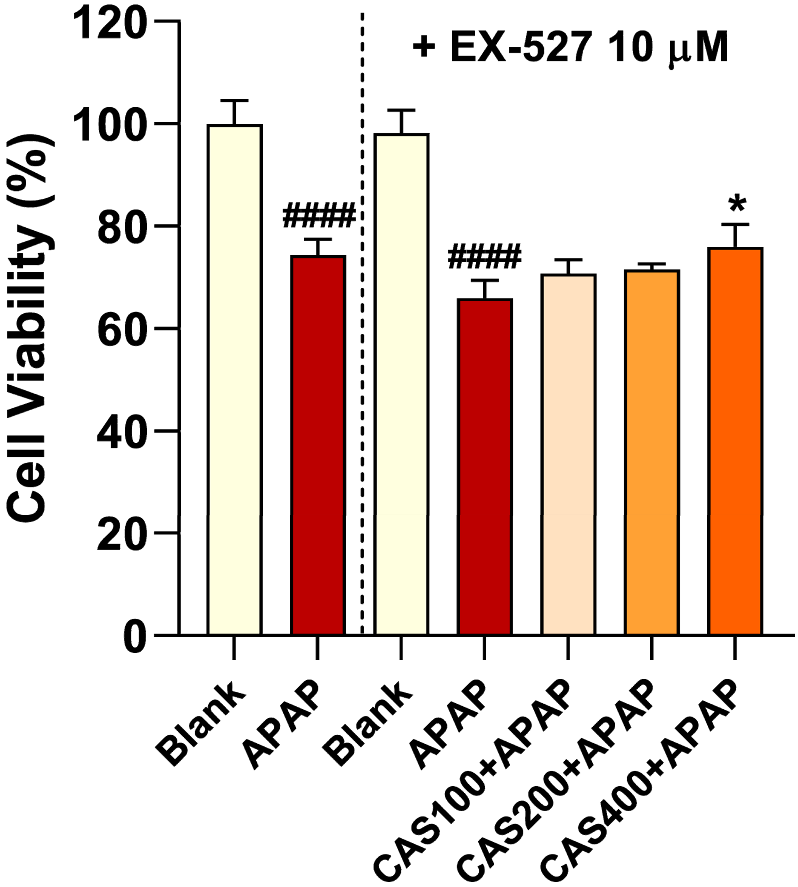

Figure S2. Cell viability of primary hepatocytes pretreated with CAS (100, 200, and 400 μg/mL) followed by subsequent exposure to Sirt1 inhibitor EX-527 with APAP for 24 h and determined by CCK-8 ($n = 4$). The data are expressed as the mean ± SEM and were analyzed via one-way analysis of variance with Tukey's post-hoc test. Significant differences are indicated as follows: #### $p < 0.0001$ vs. blank group; * $p < 0.05$ vs. APAP group.

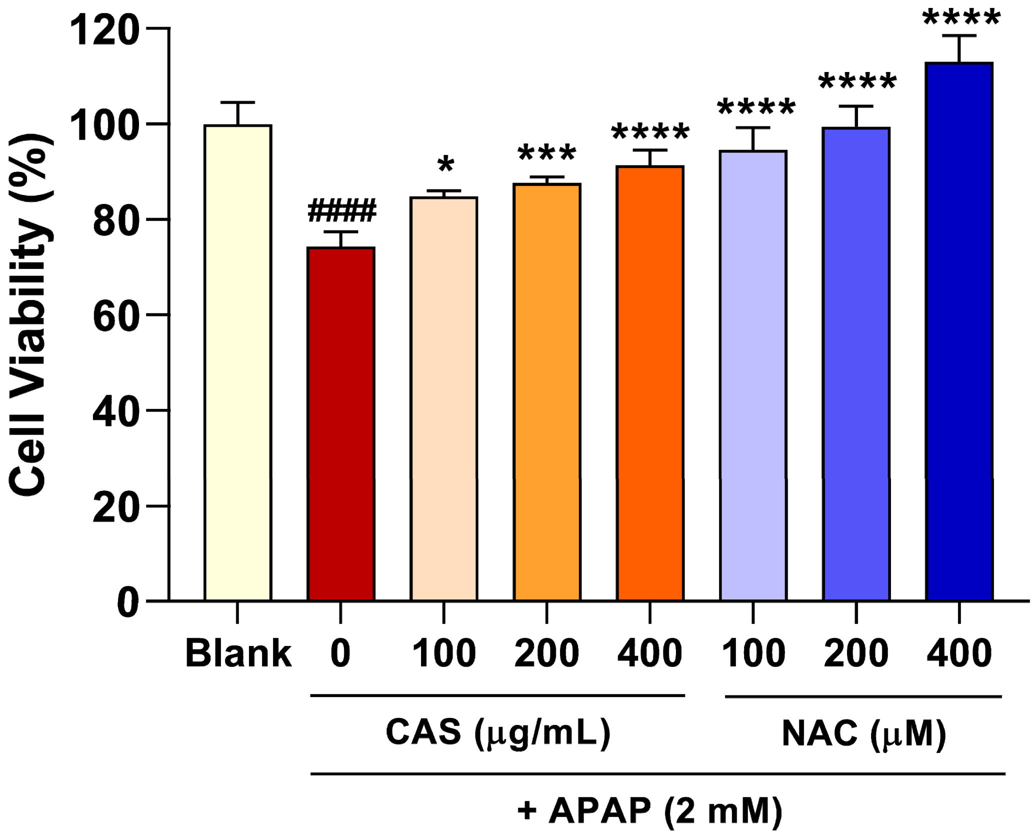

Figure S3. Cell viability of primary hepatocytes pretreated with CAS (100, 200, and 400 μg/mL) followed by subsequent exposure to ROS inhibitor NAC (100, 200, and 400 μM) with APAP for 24 h and determined by CCK-8 (n = 4). The data are expressed as the mean ± SEM and were analyzed via one-way analysis of variance with Tukey's post-hoc test. Significant differences are indicated as follows: #### p < 0.0001 vs. blank group; * p < 0.05, *** p < 0.001, and **** p < 0.0001 vs. APAP group.
